# The Orphan Nuclear Receptor SHP Is a Positive Regulator of Osteoblastic Bone Formation

**DOI:** 10.1359/jbmr.090718

**Published:** 2009-07-13

**Authors:** Byung-Chul Jeong, Yong-Soo Lee, In-Ho Bae, Chul-Ho Lee, Hong-In Shin, Hyun Jung Ha, Renny T Franceschi, Hueng-Sik Choi, Jeong-Tae Koh

**Affiliations:** 1 Dental Science Research Institute, School of Dentistry, Chonnam National University Gwangju, Republic of Korea; 2 Hormone Research Center, School of Biological Sciences and Technology, Chonnam National University Gwangju, Republic of Korea; 3 Korea Research Institute of Bioscience and Biotechnology Daejeon, Republic of Korea; 4 Department of Oral Pathology and IHBR, School of Dentistry, Kyungpook National University Daegu, Republic of Korea; 5 Department of Biochemistry, Biotechnology Research Institute, School of Life Sciences, Chungbuk National University Cheongju, Republic of Korea; 6 Department of Periodontics and Oral Medicine, University of Michigan School of Dentistry Ann Arbor, MI, USA

**Keywords:** Orphan nuclear receptor, small heterodimer partner (SHP), osteoblast differentiation, bone morphogenetic protein (BMP), Runx2

## Abstract

The orphan nuclear receptor small heterodimer partner (SHP; NR0B2) interacts with a diverse array of transcription factors and regulates a variety of cellular events such as cell proliferation, differentiation, and metabolism. However, the role of SHP in bone formation has not yet been elucidated. SHP expression is significantly increased during osteoblast differentiation, and its expression is partially regulated by bone morphogenetic protein 2 (BMP-2), which plays an important role in bone formation. In our study, inhibition of SHP expression significantly repressed BMP-2-induced osteoblast differentiation and ectopic bone formation. In accordance with these in vitro and in vivo results, osteoblast differentiation in *SHP*^−/−^ mice primary osteoblasts was significantly repressed, and the mice showed decreased bone mass resulting from decreased numbers of osteoblasts. Finally, SHP physically interacts and forms a complex with runt-related transcription factor 2 (Runx2) on the osteocalcin gene promoter, and overexpression of SHP increased Runx2 transactivity via competition with histone deacetylase 4 (HDAC4), an enzyme that inhibits DNA binding of Runx2 to its target genes. Taken together, these results indicate that SHP acts as a novel positive regulator of bone formation by augmenting osteoblast differentiation through regulation of the transcriptional activity of Runx2. © 2010 American Society for Bone and Mineral Research

## Introduction

The atypical orphan nuclear receptor (NR) SHP is a versatile protein with broad cellular functions. Structurally, SHP has a putative ligand-binding domain (LBD) that lacks a classic DNA-binding domain (DBD) and harbors two functional LXXLL-like motifs, which are typical of NR-binding proteins.(1) SHP interacts with various nuclear receptors and transcription factors, such as the estrogen receptor (ER), peroxisome proliferator–activated receptor (PPAR), retinoic acid receptor (RAR), liver X receptor (LXR), Nur77, c-Jun, Smad, histone deacetylase 1 (HDAC1), and the Swi/Snf/mSin3a corepressor complex. With the exception of PPAR, SHP acts to downregulate the transcriptional activity of these factors.(2,3) These findings suggest that as a transcriptional coregulator, SHP participates in a complex regulatory network comprised of a variety of NRs and transcription factors. Recently, studies have focused on the role of SHP in metabolism or homeostasis.(4–7) However, its function on bone metabolism has not yet been described.

Bone formation is a well-orchestrated process of lineage-specific differentiation events.(8) Osteoblasts, which play pivotal roles in bone formation, are derived from pluripotent mesenchymal stem cells that have the capacity to differentiate into myocytes, adipocytes, and chondrocytes.(9) These stem cells can differentiate into mature osteoblasts that possess the necessary components to form bone matrix and allow subsequent mineralization. Osteoblast differentiation is controlled by various hormones and cytokines, such as bone morphogenetic proteins (BMPs), and multiple transcription factors such as Runx2, Osx, Dlx5, Msx2, Twist, AP1 (Fos/Jun), Krox-20, Sp3, and ATF4.(10,11) Among these, BMPs are the primary regulators of osteoblast differentiation.(12) As a member of the transforming growth factor β (TGF-β) superfamily, the signal transduction by BMPs occurs through the activation of serine/threonine kinase receptors, which are classified as either type I or type II. After ligand binding, they form a heterotetrameric activated receptor complex and relay the signal from the receptor to target genes in the nucleus.

Runx2, also called Cbfa1, is a member of the runt domain gene family.(13) These proteins play a major role in osteoblast differentiation by promoting the differentiation of undifferentiated mesenchymal cells into osteoblasts through regulation of various factors such as type I collagen (Col I), osteopontin (OPN), bone sialoprotein (BSP), and osteocalcin (OC).(14) Runx2 has been shown to induce alkaline phosphatase (ALP) activity, expression of bone matrix protein genes, and mineralization in immature mesenchymal cells and osteoblastic cells in vitro.(15–17) During osteoblast differentiation, Runx2 interacts with diverse transcription factors(18) and recruits both coactivators (e.g., p300, HES-1, and YAP) and corepressors (e.g., TLE, mSin3a, and HDACs) to form a complex on its target promoter.(19–22)

Recently, it has been reported that various NRs are involved in osteoblast differentiation and bone formation. Activation of androgen receptors stimulates BSP gene transcription via cAMP response element (CRE) and AP1/glucocorticoid response elements (GRE).(23) Estrogen prevents bone loss via ERα,(24) and estrogen receptor–related receptor α (ERRα) regulates osteopontin expression through a noncanonical ERRα response element.(25) In this study we investigated the effects of the orphan nuclear receptor, SHP in osteoblast differentiation, and our results show that SHP promotes osteoblast differentiation and bone formation via regulation of Runx2 transactivity.

## Materials and Methods

### Plasmid constructions

The reporter constructs, −1.3 kb *OG2-Luc* and 6×*OSE-Luc*, were kindly provided by Dr RT Fransceschi (University of Michigan School of Dentistry, Ann Arbor, MI, USA). The SHP-Luc construct was described previously.(26) Runx2 expression constructs were kindly provided by Dr KY Lee (Chonnam National University, Republic of Korea). The SHP expression construct was described previously.(27) pcDNA3/HA-ALK2, −3, −5, and −6 constructs were kindly provided by Dr T Imamura **(**JFCR Cancer Institute, Japan). The pBJ5.1/Flag-HDAC3 and -4 constructs were kindly provided by Dr H Kook (Chonnam National University, Republic of Korea). The HDAC4 siRNA constructs were constructed by ligation of a 60-mer double-stranded oligonucleotide containing 5'-AATGTACGACGCCAAAGAT-3' of the HDAC4 cDNA sequence into the pSUPER vector digested with Bgl II and Xho I.

### Preparation of primary osteoblasts

Calvariae were isolated from 10-day-old neonatal mice and digested with 0.1% collagenase (Roche, Germany) at 37°C for 30 minutes. The digested calvariae were sequentially digested four times.(30) The last fractions were collected and used as primary osteoblasts.

### Cell culture and induction of osteoblast differentiation

C2C12 cells were maintained in DMEM (Hyclone, Logan, UT, USA), and C3H10T1/2 and MC3T3-E1 cells and primary osteoblasts were maintained in α-minimal essential medium (α-MEM, Gibco, Grand Island, NY, USA) supplemented with 10% fetal bovine serum (FBS, Hyclone) and antibiotics (Hyclone). Differentiation of osteoblasts was induced by 200 ng/mL recombinant human bone morphogenetic protein 2 (BMP-2, R&D Systems, Minneapolis, MN, USA), 50 µg/mL ascorbic acid (AA), and 5 mM β-glycerophosphate (β-GP).

### Transient transfection assay

Transient transfections using Lipofectamine Plus (Invitrogen, Carlsbad, CA, USA) were carried out as described previously.(28) As an internal control, cytomegalovirus (CMV) β-galactosidase plasmid was cotransfected in each transfection experiment, and luciferase activity was normalized to β-galactosidase activity.

### RT-PCR analysis

RT-PCR was performed using 0.8 µg of total RNA. Each reaction consisted of initial denaturation at 94°C for 1 minute followed by three-step cycling: denaturation at 94°C for 30 seconds, annealing at a temperature optimized for each primer pair for 30 seconds, and extension at 72°C for 30 seconds. After the requisite number of cycles (25 to 27 cycles), the reactions underwent a final extension at 72°C for 5 minutes. Primer sequenses were SHP, forward (F) 5′-CTTCCTCAGGAACCT-3′ and reverse (R) 5′-CCCAGTGAGCCTCCT-3′; OC, (F) 5′-CTCCTGAGAGTCTGACAAAGCCTT-3′ and (R) 5′-GCTGTGACATCCATTACTTGC-3′; ALP, (F) 5′-GATCATTCCCACGTTTTCAC-3′ and (R) 5′-TGCGGGCTTGTGGGACCTGC-3′; BSP, (F) 5′-ACACTTACCGAGCTTATGAGG-3′ and (R) 5′-TTGCGCAGTTAGCAATAGCAC-3′; Runx2, (F) 5′-GAGGGCACAAGTTCTATCTG-3' and (R) 5′-CGCTCCGGCCCACAAATCTC-3′; Col I, (F) 5′-TCTCCACTCTTCTAGGTTCCT-3′ and (R) 5′-TTGGGTCATTTCCACATGC-3′; Osx, (F) 5′-GAAAGGAGGCACAAAGAAG-3′ and (R) 5′-CACCAAGGAGTAGGTGTGT-3′; OSCAR, (F) 5′-CTGCTGGTAACGGATCAGCTCCCCAGA-3′ and (R) 5′-CCAAGGAGCCAGAACCTTCGAAACT-3′; TRAP, (F) 5′-CTGGAGTGCACGATGCCAGCGACA-3′ and (R) 5′-TCCGTGCTCGGCGATGGA CCAGA-3′; NFATc1, (F) 5′-CTCGAAAGACAGCACTGGAGCAT-3′ and (R) 5′-CGGCTGCCTTCCGTCTCA TAG-3′; and GAPDH, (F) 5′-ACCACAGTCCATGCCATCAC-3′ and (R) 5′-TCCACCCTGTTGCTGTA-3′. Although all results obtained from RT-PCR are, by definition, not quantitative, apparent quantitative PCR analysis was done at around 25 cycles with relatively high linearity using primer for the housekeeping gene glyceraldehydes-3-phophate dehydrogenase (*GAPDH*) toward the possible comparative reference.

### Chromatin immunoprecipitation (ChIP) assay

C2C12 or 293T cells were cotransfected with the designated expression constructs and –1.3 kb *OG2-Luc* reporter gene. Then, 48 hours after transfection, ChIP assays were performed as described previously.(28) The primer sequences for the Runx2-binding region of the *OG2* were as follows: (F) 5′-GAGGACATTACTGAACAC-3′ and (R) 5′-CAGTGGGTCAAACCCAAA -3′.

### Recombinant adenovirus preparation and virus infection

The adenoviral vectors for full-length SHP (Ad-SHP) and SHP siRNA (Ad-siSHP) were described previously.(27,28) Ad-BMP and Ad-Runx2 were described previously.(29) Briefly, cells were infected at the designated multiplicity of infection (MOI) using indicated viruses in no serum for 4 hours. Following viral infection, an equivalent volume of medium containing 4% FBS was added, and cells were incubated for an additional 24 hours before osteogenic medium treatment containing AA (50 µg/mL) and β-GP (5 mM) in the presence of BMP-2 (200 ng/mL).

### Alkaline phosphatase activity and osteocalcin production assays

ALP activity was measured in cell layers using *p*-nitrophenyl phosphate substrate as described previously.(31) ALP activity was normalized with DNA that was measured by a PicoGreen dsDNA quantitation kit (Molecular Probes, Eugene, OR, USA). The amount of osteocalcin (OC) secreted into the culture medium was determined using a mouse OC ELISA kit (Biomedical Technologies, Inc., Stoughton, MA, USA) according to the manufacturer's instructions.

### Alizarin red staining

Cells were fixed with 70% ethanol, rinsed three times with deionized water, and then treated with a 40 mM alizarin red stain (AR-S) solution (pH 4.2) for 10 minutes. Stained cultures were photographed and then quantitatively extracted using 10% (w/v) cetylpyridinium chloride (CPC) in 10 mM sodium phosphate (pH 7.0) for 15 minutes.

### Osteoclast differentiation

Murine osteoclasts were prepared from bone marrow cells and cultured in α-MEM containing 10% FBS with M-CSF (5 ng/mL) for 16 hours. Nonadherent cells were harvested and cultured for 3 days with M-CSF (30 ng/mL). Floating cells were removed, and the attached cells were used as osteoclast precursors [bone marrow–derived monocyte/macrophage lineage cells (BMMs)]. To generate osteoclasts, BMMs were cultured with M-CSF (30 ng/mL) and indicated amounts of RANKL for 3 days. Cultured cells were fixed and stained for tartrate-resistant acid phosphatase (TRAP), and TRAP-positive multinuclear cells (TRAP+ MNCs) containing more than three nuclei were counted by Leica DMIRB microscope equipped with an N Plan 10×/0.25 numerical aperture objective lens (Leica, Wetzlar, Germany). Images were obtained using a Leica IM50 camera and Leica IM 4.0 software (Leica, Cambridge, UK).

### X-ray and micro-computed tomographic (µCT) scanning

C57BL/6J mice (male, 6 weeks old) were injected with a fixed dose of 5 × 10^10^ PN (particle number) of the designated adenoviral constructs in the thigh muscle: mock virus (*n* = 3), Ad-BMP-2 (*n* = 3), Ad-siSHP (*n* = 3), and Ad-BMP-2 plus Ad-siSHP (*n* = 3) diluted in PBS. Ectopic bone formation was monitored by radiographic apparatus (Hi-Tex, Osaka, Japan) at 35 kVp and 400 µA (2D). Microarchitecture of the femoral and tibial trabecular bone was investigated using µCT (Skyscan 1172, Skyscan, Kontich, Belgium) in cone-beam acquisition mode. The X-ray source was set at 50 kV and 200 A with a pixel size of 17.09 µm. Exposure time was 1.2 seconds. Four-hundred and fifty projections were acquired over an angular range of 180 degrees (angular step of 0.4 degree). The image slices were reconstructed using 3D CT analyzer software (CTAN, Skyscan). Trabecular morphometry was characterized by measuring the bone volume fraction (BV/TV), trabecular thickness (Tb.Th), trabecular number (Tb.N), and trabecular separation (Tb.Sp). For static histomorphometry, the tibia from each mouse was removed and fixed in 4% paraformaldehyde in PBS at 4°C, decalcified, dehydrated in progressive concentrations of ethanol, cleared in xylene, and embedded in paraffin. Quantification of osteoblasts and osteoclasts was performed in paraffin-embedded tissue, as described previously.(32)

### Animals

*SHP* null (*SHP*^−/−^) mice on a C57BL/6 and 129/Sv mixed background were backcrossed with C57BL/6 mice 10 times to generate congenic C57BL/6 *SHP*^−/−^ mice, as described previously.(33) Age-matched male *SHP*^−/−^ congenic and wild-type (WT) C57BL/6 mice were used throughout this study. All animal studies were carried out under the guidelines of the Chonnam National University Animal Care and Use Committee.

### Statistical analysis

All experiments were repeated at least three times, and Student's *t* test was used to measure statistically significant differences among groups. Unless otherwise indicated, experimental data are expressed as means ± SD of triplicate independent samples.

## Results

### SHP gene expression is increased during osteoblast differentiation and regulated by BMP-2

**Fig. 1:**
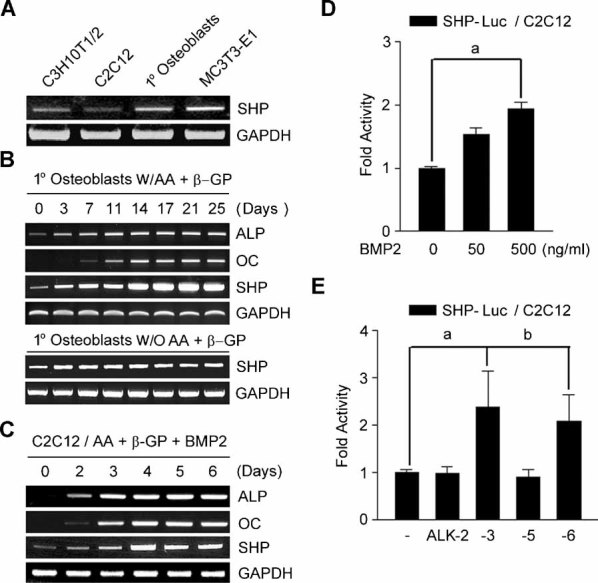
*SHP* gene expression during osteoblast differentiation and SHP promoter activity induced by BMP-2. (*A*) Endogenous expression of SHP in various progenitor cells. Total RNAs were isolated from the 3-day-cultured cells and used for RT-PCR with mouse SHP and GAPDH primers. (*B*) mRNA expression of osteoblast-specific genes and SHP in primary (1°) osteoblast differentiation. Mouse primary osteoblasts were maintained for 25 days in α-MEM containing AA (50 µg/mL) and β-GP (5 mM). At the designated time points, cells were harvested for total RNA isolation, and RT-PCR was performed with the indicated primers. The lower figure demonstrates the endogenous expression of SHP in the absence of AA and β-GP as a control. (*C*) mRNA expression of ALP, OC, and SHP during osteoblast differentiation by BMP-2. C2C12 cells were cultured for 6 days in DMEM containing 200 ng/mL of BMP-2 in the presence of AA and β-GP. At the designated time points, total RNAs were isolated and used for RT-PCR. (*D*, *E*) SHP promoter activity by BMP-2 and constitutive active forms of the BMP receptor. C2C12 cells were transfected with 100 ng of *SHP-Luc* reporter plasmid and 50 ng of pCMV-β-galactosidase as an internal control in the presence of the indicated amounts of recombinant human BMP-2 (*D*) or 100 ng of pcDNA3/HA-ALK2, -3, -5, and -6 (*E*). Reporter assays were performed as described in “Materials and Methods.” a, b = *p* < .03.

SHP is expressed in various tissues and involved in a complex regulatory network comprised of a variety of NRs and transcription factors.(2,3) To assess whether SHP might play a functional role in bone metabolism, the endogenous expression of SHP was examined in various progenitor cells. As shown in Fig. 1A, SHP is expressed in C3H10T1/2 pluripotent mesenchymal cells, C2C12 myoblastic cells, mouse primary osteoblasts, and MC3T3-E1 preosteoblastic cells. During the osteoblastic differentiation process, the expression levels of ALP and OC, which are typical osteoblast differentiation markers, were increased.(34) Importantly, the expression pattern of SHP was similar to that of ALP and OC after treatment with ascorbic acid (AA) and β-glycerophosphate (β-GP), which are classic inducers of osteoblast differentiation (Fig. 1B). The TGF-β superfamily member BMP-2 plays a central role during osteoblast differentiation.(12) To investigate the expression level of SHP during BMP-2-dependent osteoblasts differentiation, C2C12 cells were treated with BMP-2 in combination with AA and β-GP for 6 days. As shown in Fig. 1C, the addition of BMP-2 significantly induced the expression of SHP, in conjunction with other osteoblast differentiation markers, at a much earlier time point. These results indicate that the expression of SHP is temporally regulated during osteoblast differentiation, suggesting that SHP may play a specific role in bone development.

As shown in Fig. 1C, the expression level of SHP also was increased by BMP-2, suggesting that BMP-2 is a potential regulator of *SHP* gene expression. Therefore, we examined SHP promoter activity in response to either treatment with exogenous BMP-2 or expression of various activated BMP receptors in C2C12 cells. As shown in Fig. 1D, SHP promoter reporter activity was increased by treatment with BMP-2 in a dose-dependent manner. To determine whether the increased SHP promoter activity is dependent on BMP signaling, constitutively active forms of TGF-β superfamily such as type IA activin receptor (ALK2), BMP type IA receptor (ALK3), TGF-β type I receptor (ALK5), and BMP type IB receptor (ALK6) were cotransfected with a SHP promoter reporter construct. The data showed that only ALK3 and -6, which are typical BMP receptors, activated SHP promoter activity (see Fig. 1E), suggesting that BMP-2 is a potential regulator of SHP.

### Inhibition of SHP expression represses expression of BMP2–induced osteoblast differentiation markers

**Fig. 2:**
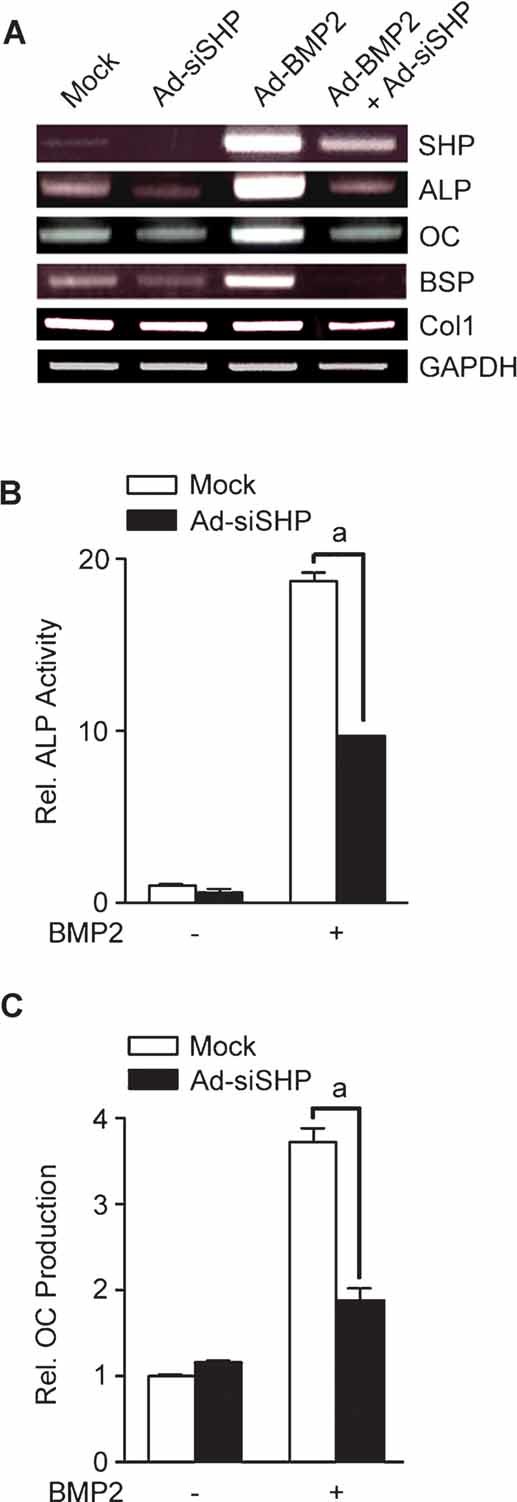
BMP-2-induced osteoblast-specific gene expression following inhibition of SHP expression. (*A*) mRNA expression of osteoblast-specific genes by the inhibition of SHP expression. C3H10T1/2 cells were infected with the indicated adenovirus at an MOI of 100. Mock virus was used as a control. Then, 48 hours after infection, the cells were harvested, and total RNA was prepared for RT-PCR with designated primers. (*B*, *C*) BMP-2-induced ALP activity and OC production by the inhibition of SHP expression. C2C12 cells were infected with Mock virus or Ad-siSHP in the presence or absence of Ad-BMP-2. Three days later, cells were harvested, and the lysates and culture medium were used for ALP activity and OC production assays, respectively, as described in “Materials and Methods.” a = *p* < .001.

BMP-2 promotes osteoblast differentiation via regulation of the expression of various osteoblast-specific genes.(35,36) To determine if there is a direct effect of SHP on BMP-2-induced osteoblast differentiation, *SHP* gene expression was inhibited by infecting C3H10T1/2 cells with an adenoviral vector expressing SHP small interfering RNA (Ad-siSHP). The expression of osteoblast differentiation markers then was observed in the presence of BMP-2. As shown in Figure 2A, adenovirus-mediated overexpression of BMP-2 in control cells induced the expression of osteoblast-specific genes such as *ALP*, *OC*, *BSP*, and *Col I* in conjunction with an increase in SHP expression, whereas expression of these differentiation marker genes was significantly repressed by Ad-siSHP. Interestingly, the inhibition of SHP also decreased the marker gene expression without BMP-2. As a functional analysis, we further confirmed the changes in ALP activity and OC production by Ad-siSHP-mediated inhibition of SHP expression in C2C12 cells. As shown in Figure 2B and C, BMP-2-induced ALP activity and OC production were significantly reduced by Ad-siSHP. Taken together, these results indicate that inhibition of *SHP* gene expression results in decreased expression of *ALP*, *OC*, *BSP* and *Col I*, all of which are involved in the regulation of osteoblast differentiation.

### SHP regulates BMP-2-induced mineralization and ectopic bone formation

**Fig. 3:**
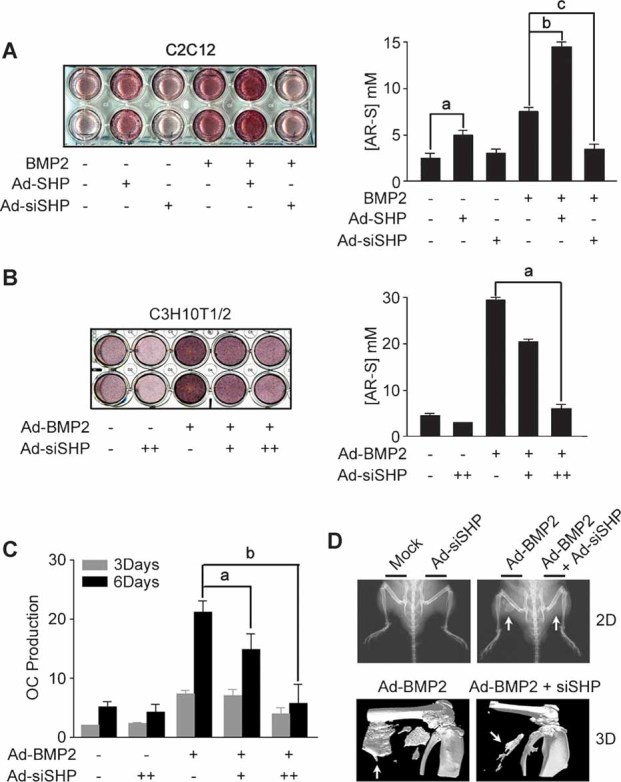
The effect of SHP on BMP-2-induced mineralization and ectopic bone formation. (*A*) SHP increased mineralized nodule formation induced by BMP-2. C2C12 cells were infected with the indicated adenovirus at an MOI of 100. The, 24 hours after infection, the medium was changed to a mineralizing medium containing AA and β-GP in the presence or absence of BMP-2 for 6 days. (*B*) Ad-siSHP decreased BMP-2-induced mineralized nodule formation. C3H10T1/2 cells were infected with the indicated adenovirus at 50 (+) to 100 (++) MOI. Then, 24 hours after infection, the medium was changed to a mineralizing medium. During the cell culture, the medium was changed every other day. After 7 days, cells were fixed and stained with alizarin red stain (AR-S) solution, and the AR-S concentration was measured as described in “Materials and Methods.” The right panels of (*A*) and (*B*) depict the produced AR-S concentrations. (*C*) Ad-siSHP decreased BMP-2-induced OC production. At 3 and 6 days after adenoviral infection, the culture medium was harvested (by the same method as in the experiment described in panel *B*), and the amounts of OC secreted into the culture medium were measured as described in “Materials and Methods.” a, b, c = *p* < .01. (*D*) Decreased BMP-2-induced ectopic bone formation by the inhibition of SHP expression. 5 × 10^10^ PNs (particle numbers) of each adenovirus was injected into thigh muscle of mice. Five weeks after injection, ectopic bone formation was analyzed by X-ray (2D) or µCT (3D). The white arrow indicates newly formed bone tissue.

Extracellular matrix mineralization is the most important phenomenon in bone formation and is regulated by various osteogenic factors.(37) The preceding results revealed that SHP regulates osteoblast differentiation. To verify the functional influences of SHP on BMP-2-induced mineralization, we infected C2C12 with Ad-SHP or Ad-siSHP in the presence or absence of BMP-2 and then assessed the amount of mineralization by alizarin red staining. As shown in Figure 3A, adenovirus-mediated SHP overexpression moderately increased mineralized nodule formation even in the absence of BMP-2, whereas overexpression of SHP in combination with the addition of BMP-2 dramatically increased the amount of mineralization compared with BMP-2 alone. However, BMP-2-induced mineralization was significantly decreased by inhibition of SHP expression, even in the presence of added BMP-2. These results suggest that SHP is physiologically involved in BMP-2-induced mineralization. To confirm this result in a different cell line, we examined the effects of SHP expression on BMP-2-induced mineralized nodule formation in C3H10T1/2 cells. As shown in Figure 3B, Ad-BMP-2 dramatically increased mineralization, whereas inhibition of SHP expression with Ad-siSHP repressed the mineralization in a dose-dependent manner. In addition, BMP-2-induced OC production also was significantly decreased by Ad-siSHP during cell culture (see Fig. 3C). These results indicate that SHP has a functional role in BMP-2-induced mineralization. It is well reported that subcutaneous or intramuscular administration of BMP-2 induces ectopic cartilage and bone formation.(12) To determine the effect of SHP on intramuscular bone formation in vivo, BMP-2-induced ectopic bone formation was analyzed in conjunction with Ad-siSHP intramuscular injection. Radiographic (X-ray, 2D) and µCT (3D) analysis revealed that adenovirus-mediated BMP-2 expression profoundly induced ectopic bone formation in the thigh muscle, whereas inhibition of SHP expression significantly decreased the bone formation (see Fig. 3D). Taken together, these results indicate that SHP regulates the expression of osteoblast-specific genes and the mineralization of potent osteoblastic cells that contribute to substantial bone formation.

### Repressed osteoblast differentiation in SHP^−/−^ mouse

**Fig. 4:**
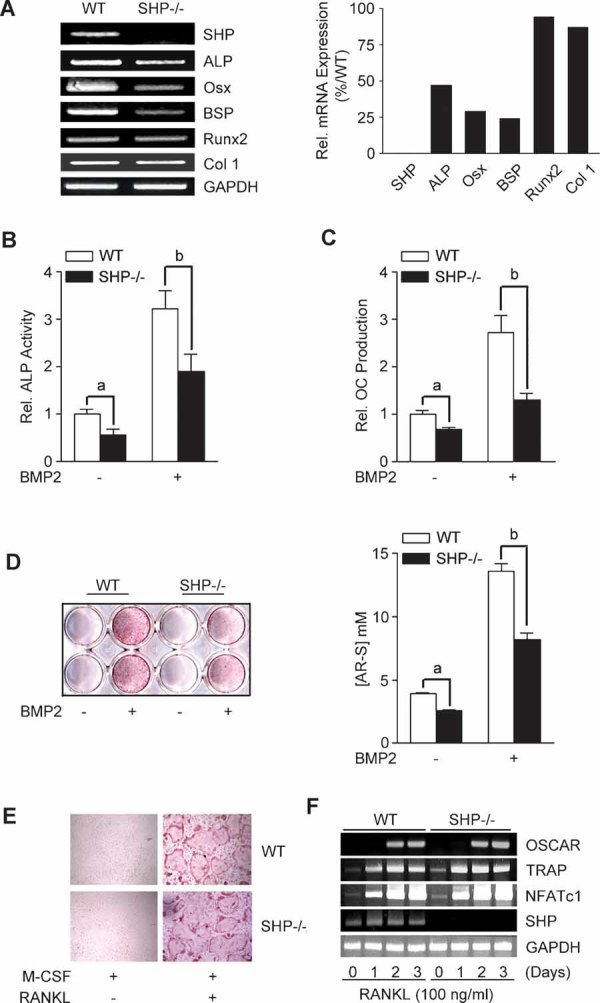
Repressed osteoblast differentiation in *SHP*^−/−^ mouse. (*A*) Endogenous mRNA expression of osteoblast-specific genes in WT and *SHP*^−/−^ mouse primary osteoblasts. Total RNAs were isolated and used for RT-PCR with the indicated primers. The right panel depicts the relative mRNA expression levels of each gene from *SHP*^−/−^ mice compared with WT mice (% WT). (*B–D*) BMP-2-induced ALP activity (*B*), OC production (*C*), and mineralized nodule formation (*D*) in WT and *SHP*^−/−^ mice. The primary osteoblasts from WT and *SHP*^−/−^ mice were maintained in α-MEM containing AA and β-GP in the presence or absence of BMP-2 for 9 days (for ALP and OC) or 20 days (for AR-S). a, b = *p* < .01. (*E*) TRAP staining of mouse bone marrow cells induced by M-CSF and RANKL. Isolated bone marrow cells from WT and *SHP*^−/−^ mice were cultured in α-MEM containing 10% FBS with M-CSF (5 ng/mL) for 16 hours. To generate osteoclasts, the cells were cultured with M-CSF and RANKL (100 ng/mL). After 3 days in culture, the cells were fixed and stained for TRAP, and TRAP-positive multinuclear cells (TRAP^+^ MNCs) containing more than three nuclei were counted under the microscope. (*F*) mRNA expressions of osteoclast-specific marker genes. Duplicate cultures, as described in panel (*E*), were prepared. At designated time points, the cells were harvested, and then total RNAs were prepared for RT-PCR.

To explore the physiologic effect of SHP in an animal model, we observed the endogenous expression profile of osteoblast marker genes and osteoblast differentiation in the *SHP*^−/−^ mouse. As shown in Figure 4A, the expression levels of ALP, BSP, and osterix (OSX) were significantly decreased in *SHP*^−/−^ mouse calvarial cells compared with WT mice. However, the expression levels of Col I and Runx2, a major transcription factor for osteoblast differentiation, were not significantly changed in *SHP*^−/−^ mice, suggesting that SHP does not participate in the regulation of Runx2 or Col I expression. To determine the effect of BMP-2 on *SHP*^−/−^ mouse primary osteoblasts, isolated calvarial cells were treated with AA and β-GP to induce osteoblast differentiation in the presence or absence of BMP-2. As shown in Figure 4B and C, BMP-2-induced ALP activity and OC production were significantly decreased in *SHP*^−/−^ mice compared with WT mice. In addition, mineralized nodule formation by BMP-2 was also decreased in *SHP*^−/−^ mice (see Fig. 4D), suggesting that the absence of SHP causes a defect in osteoblast differentiation. These results indicate that endogenous SHP is substantially involved in osteoblast differentiation and mineralization. Osteoblast and osteoclast differentiations are sophisticated and well-orchestrated processes, and proper balance between both processes is critical for proper bone metabolism.(38) To examine whether SHP is involved in osteoclast differentiation, we examined the expression of osteoclast marker genes and tartrate resistant acid phosphatase (TRAP) staining from *SHP*^−/−^ mouse bone marrow cells that are induced by receptor activator of NF-κB ligand (RANKL), a key inducer of osteoclast differentiation. As shown in Figure 4E and F, there was no clear difference in the typical morphology of osteoclasts and the expression patterns of marker genes such as osteoclast-associated receptor (*OSCAR*), *TRAP*, and nuclear factor of activated T cells (*NFATc1*) between WT and *SHP*^−/−^ mouse bone marrow cells. These results indicate that SHP has no specific role in osteoclast differentiation and rather that it has more critical functions on osteoblast differentiation in bone metabolism.

### Decreased bone mass in the SHP^−/−^ mouse

**Fig. 5:**
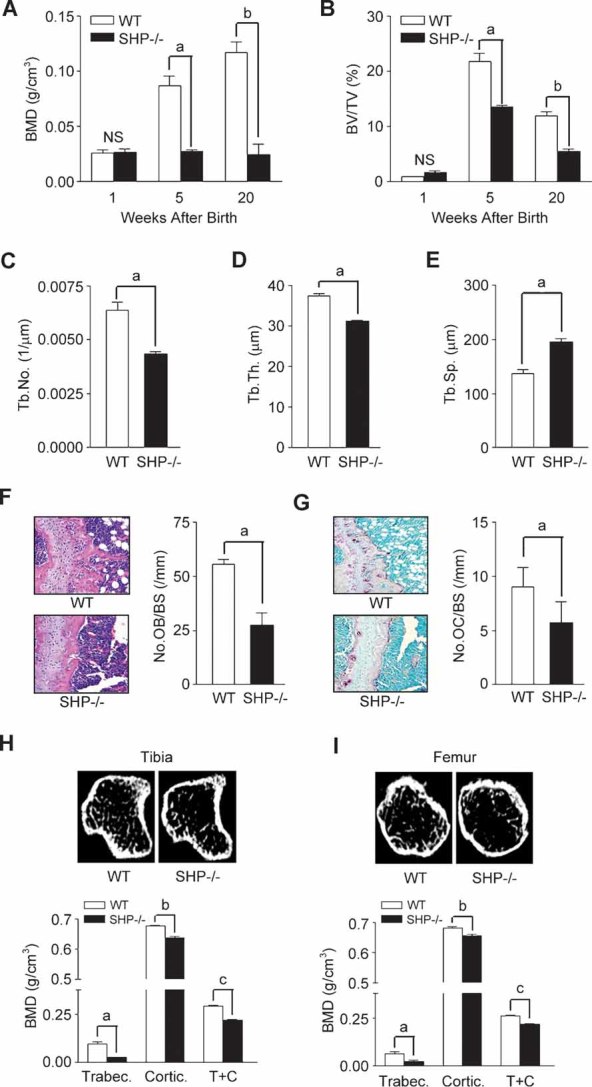
Decreased bone mass in *SHP*^−/−^ mice. (*A*, *B*) Bone mineral density (BMD) and bone volume/total volume (BV/TV) of tibiae from 1-, 5-. and 20-week-old male WT (*n* = 6) and *SHP*^−/−^ mice (*n* = 6) using µCT, respectively. (*C–E*) Trabecular bone number (Tb. No.), trabecular thickness (Tb. Th), and trabecular separation (Tb. Sp) of tibiae. (*F*, *G*) Histomorphometric analysis of bone formation (*left*) and quantification of osteoblasts and osteoclasts per bone surface (*right*), respectively. (*H*, *I*) Representative cross sections of the tibia and femur shaft (*top*) and the bone density of the cortical and trabecular region (*bottom*), respectively. Six-week-old male WT and *SHP*^−/−^ mice were used in experiments C through I (*n* = 4). a, b, c = *p* < .03. NS = nonsignificant.

To investigate the impact of SHP expression on direct bone formation, we compared the bone mass of WT and *SHP*^−/−^ mice by µCT analysis and histomorphometric measurements in sections of tibiae collected at the ages of 1, 5 or 6, and 20 weeks, respectively. As shown in Figure 5A, bone mineral density (BMD) of tibiae from 5- and 20-week-old *SHP*^−/−^ mice was significantly decreased compared with WT mice. Moreover, the bone mineral volume (BV/TV) also was decreased in same-aged *SHP*^−/−^ mice (see Fig. 5B). However, there was no comparable change in bone density or BV/TV of 1-week-old *SHP*^−/−^ mice, indicating that SHP functions primarily at a relatively late stage of bone development. To investigate more detailed static bone histomorphometry, we measured each degree of trabecular bone number (Tb. No.), thickness (Tb. Th.), and separation (Tb. Sp.) in 6-week-old WT and *SHP*^−/−^ mice. Similar to the findings in femurs, *SHP*^−/−^ mice tibiae exhibited a decrease in trabecular number and thickness as well as an increase in trabecular bone separation (see Fig. 5C–E). Furthermore, the number of osteoblasts per bone surface was significantly reduced in *SHP*^−/−^ mice (see Fig. 5F). In addition, the growth plate of the *SHP*^−/−^ mice had considerably fewer chondrocytes than WT mice, which have an elongated region of hypertrophic cartilage (see Fig. 5F, *left panel*). Interestingly, the histomorphometric analysis showed that the number of osteoclasts also was reduced in *SHP*^−/−^ mice (see Fig. 5G). 3D µCT measurements especially revealed a significant decrease in trabecular thickness in *SHP*^−/−^ mice (see Fig. 5H and I). Collectively, these results suggest that SHP is involved in determining substantial bone architecture.

### SHP physically interacts with Runx2 and regulates its transactivity

**Fig. 6:**
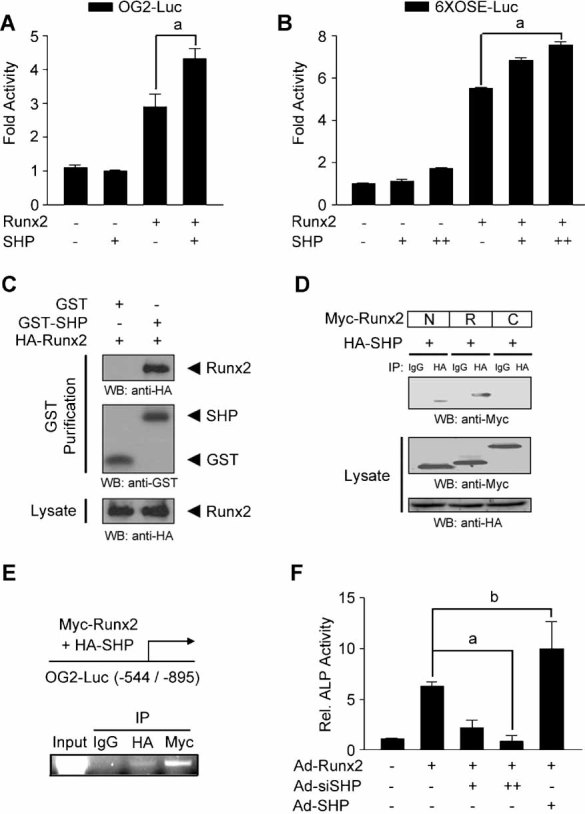
Regulation of Runx2 activity by association with SHP. (*A*, *B*) SHP increases Runx2 transactivity. C3H10T1/2 (*A*) and C2C12 (*B*) cells were cotransfected with 200 ng of the indicated luciferase reporter and 100 ng of Runx2 together with 100 ng (+) or 200 ng (++) of HA-SHP. (*C*) Physical interaction of SHP with Runx2. In vivo GST pull-down assay was performed with 293T cells as described in “Materials and Methods.” (*D*) Mapping the SHP interaction domains of Runx2. 293T cells were cotransfected with Myc-tagged different domains of Runx2 (N: 1–92; R: 93–220; C: 221–513 amino acids) and HA-SHP expression vectors. Immunoprecipitation (IP) assay was performed with anti-HA and control IgG. (*E*) Complex formation of Runx2 and SHP on osteocalcin gene (OG2) promoter region. 293T cells were cotransfected with 3 µg of HA-SHP and Myc-Runx2 together with OG2 promoter. Then 48 hours after transfection, whole cell lysates were prepared and subjected to IP using respective antibodies, and the isolated DNA was analyzed by PCR. Primers were designed to amplify the Runx2-binding region on the OG2 promoter. Ten percent of the input whole cell lysate was used as the input control. (*F*) The effects of SHP on the Runx2-mediated ALP activity. C3H10T1/2 cells were infected with the indicated adenovirus at an MOI of 100. Then, 24 hours after infection, the medium was changed to the osteogenic medium. Eight days later, ALP activity was determined as described previously. a, b = *p* < .01.

To determine the mechanism by which SHP promotes osteoblast differentiation, we examined the effect of SHP on the transactivity of Runx2 because BMP-2-Runx2 cascade is important for the differentiation of mesenchymal stem cells into the osteoblast lineage.(18) Moreover, bone-specific expression of OC is regulated principally by Runx2.(39) To examine whether SHP affects Runx2 function, the effect on Runx2 transactivity by SHP expression was analyzed in C3H10T1/2 cells. As shown in Figure 6A, the Runx2-dependent reporter activity of *OG2-Luc* was enhanced by overexpression of SHP. In C2C12 cells transfected with a 6×*OSE-Luc*, SHP also significantly increased Runx2 transactivity in a dose-dependant manner (see Fig. 6B). To gain insights into the mechanism by which SHP increases Runx2 transactivity, we examined the physical interaction between SHP and Runx2 using an in vivo glutathione-*S*-transferase (GST) pull-down assay. As shown in Figure 6C, SHP physically interacted with Runx2 protein, showing that SHP activates Runx2 transactivity by direct interaction. To determine the interacting region of Runx2, we performed immunoprecipitation (IP) assay with different fragments of Runx2 proteins. As shown in Figure 6D, SHP strongly interacted with the Runt (R) domain, although there was weaker interaction with the amino terminus (N) of Runx2. To further examine whether there is any complex formation between SHP and Runx2 on their target gene promoter, ChIP assay was performed. As shown in Figure 6E, SHP interacted with Runx2, which bound to its target binding sequences of osteocalcin gene promoter. Consistent with these results, Ad-Runx2-mediated ALP activity was markedly repressed by the inhibition of SHP expression, whereas overexpression of SHP additively increased Runx2-dependent ALP activity (see Fig. 6F). We further examined the Runx2 protein levels in primary osteoblasts of WT and *SHP*^−/−^ mice. However, we could not detect any difference in Runx2 protein levels (data not shown). Taken together, these results indicate that SHP may regulate osteoblast differentiation through enhancement of Runx2 activity not via regulation of Runx2 protein level.

### SHP regulates Runx2 transactivity via competition with HDAC4

**Fig. 7:**
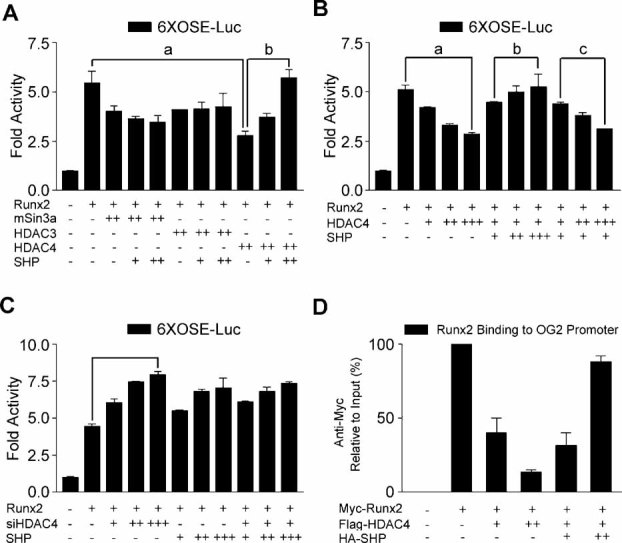
Regulation of Runx2 transactivity by SHP via competition with HDAC4. (*A*, *B*) The effects of SHP on the transcriptional corepressors of Runx2. C2C12 cells were cotransfected with 200 ng of the six copies *OSE-Luc* reporter and 100 ng of Runx2 together with 100 ng (+) or 200 ng (++) of mSin3a, HDAC3 or -4 and SHP expression vectors. (*C*) Repression of endogenous HDAC4 expression increased Runx2 transactivity. C2C12 cells were cotransfected with 200 ng of the reporter and Runx2 together with different doses of siHDAC4 and/or SHP. Reporter assay was performed as described previously. (*D*) SHP recovers the repressed DNA binding of Runx2 by HDAC4. C2C12 cells were cotransfected with 0.5 µg (+) or 1 µg (++) of Myc-Runx2, Flag-HDAC4, and HA-SHP together with 0.5 µg of OG2 promoter. Then, 48 hours after transfection, whole cell lysates were prepared and subjected to ChIP as described previously. a, b, c = *p* < .01

Previous studies reported that various coactivators or corepressors interact with Runx2 to promote or repress Runx2 transactivity.(19,21,22) To examine whether SHP has any effect on transcriptional corepressors of Runx2, we used a transient transfection assay in C2C12 cells. The selected corepressors of Runx2, such as mSin3a, HDAC3, and HDAC4, significantly repressed Runx2 transactivity (data not shown). Among the corepressors, HDAC4 was the most strongly affected by SHP, which showed a significant recovering function for the HDAC4's repressive activity (Fig. 7A). Moreover, SHP competed with HDAC4 for an influence over Runx2 transactivity (see Fig. 7B), and inhibition of endogenous HDAC4 increased Runx2 transactivity (see Fig. 7C), demonstrating that HDAC4 is a substantial corepressor of Runx2. A previous study reported that HDAC4 inhibits Runx2 from binding to its target gene, such as osteocalcin.(40) To further examine whether SHP competes with HDAC4 in vivo, we performed a ChIP assay in C2C12 cells. As shown in Figure 7D, expression of HDAC4 inhibited Runx2 DNA binding to the osteocalcin gene promoter, whereas expression of SHP relieved the repressed DNA binding of Runx2 in a dose-dependent manner. These results indicate that SHP promotes Runx2 transactivity through competition with HDAC4.

## Discussion

Osteoblast differentiation and bone matrix formation are finely tuned processes that are tightly regulated by a variety of transcription factors. Recently, it has been reported that several NRs regulate osteoblast differentiation via promotion of osteogenic gene expression.(23,25) In this study, we investigated a role of the orphan NR SHP in bone formation. In osteoblastic cells, BMP-2 significantly induced *SHP* gene expression and induction of osteoblast differentiation as well as increased SHP promoter activity. As a member of the TGF-β superfamily, BMP-2 plays a central role in osteoblast differentiation and bone formation(12) through multiple downstream pathways, including the Smad pathway, which is initiated by phosphorylation of Smad proteins by type I receptors and the mitogen-activated protein kinase (MAPK) pathway. In either case, the ultimate consequence of BMP-2 signaling is the activation of gene transcription, which promotes osteoblast differentiation and bone formation. Therefore, the expression of SHP may be regulated by a certain mediator(s) that is involved in the BMP signaling pathway. To identify the potential mediator, we examined the effects of several regulators that are activated by BMP-2, such as Smads, JNK, c-Jun, Dlx5, Msx2, Runx2, and Osx, on SHP promoter activity. However, we were unable to identify an activator of the SHP promoter construct (data not shown). Meanwhile, some of our results revealed that AA and β-GP induced SHP expression in mouse calvarial cells (see Fig. 1B) and that SHP overexpression by Ad-SHP also induced mineralization of osteoblastic cells, even in the absence of BMP-2 (see Fig. 3A). Therefore, we cannot rule out the possible existence of either a BMP-2-independent modulator or an alternative BMP-2-dependent pathway that promotes *SHP* gene expression, and the possibility has lead us to begin the search for a novel SHP regulator.

The fact that SHP expression is upregulated during osteoblast differentiation motivated us to speculate that SHP may have a positive role in bone metabolism, in contrast to its classic functions. In order to determine the endogenous role of SHP in bone differentiation, we observed BMP-2-induced osteoblast differentiation patterns in an SHP-deficient state becausee overexpression of SHP strongly augmented mineralized nodule formation induced by BMP-2 (see Fig. 3A). The data demonstrated that inhibition of SHP expression reduced the expression of osteoblast-specific marker genes, mineralization of osteoblasts, and ectopic bone formation in the mouse. These results are in agreement with the results obtained from in vivo experiments in *SHP*^−/−^ mice that revealed reduced osteoblast differentiation patterns and decreased bone mass. Interestingly, loss of SHP per se resulted in the reduction of osteogenesis regardless of BMP-2 existence. In addition, loss of SHP expression in primary osteoblasts, which show relatively high SHP expression (see Figs. 1A and 4A), reduced the expression of ALP activity and mineralization without BMP-2 (see Fig. 4B–D), but did not produce any significant changes in C2C12 cells, which show relatively low levels of SHP expression without BMP-2 (see Fig. 2B, C). This result indicates that SHP acts directly on osteogenesis with BMP-2-dependent or -independent signaling and suggest that SHP differentially regulates osteoblast differentiation in a cell-type- and a context-dependent manner.

In particular, trabecular BMDs of the tibia and femur were significantly decreased in *SHP*^−/−^ mice, much more so than in the cortical bone. These results indicate that reduced bone mass may be due to decreased trabecular BMD. Meanwhile, a lack of SHP had no significant effect on bone mass in young mice (1 week after birth), which suggests that SHP is involved in the later stages of bone development. Recent study demonstrated that BMP-2 activity is required for postnatal bone physiology.(41) Therefore, our results indicate that the potential existence of a regulator of SHP expression or activation whose activity depends on developmental cues in connection with BMP-2 and that SHP may cooperate with BMP-2 for the late developmental stage in vivo. Bone metabolism is determined by the balance between osteoblastic cell function for bone formation and osteoclastic cell function for bone resorption, and various regulators act to maintain that balance.(42,43) With regard to osteoclast differentiation, loss of SHP expression had no significant effect on cellular development or on the expression of osteoclast specific marker genes such as *OSCAR*, *TRAP*, and *NFATc1* (see Fig. 4E, F). However, our in vivo result showed that loss of SHP causes decreased osteoclast numbers as well as osteoblast numbers (see Fig. 5F, G). These results suggest that SHP has no direct effect on osteoclast differentiation, but it has an indirect effect on osteoclast number owing to the inhibition of osteoclastogenesis, which is partially regulated by mature osteoblasts.

In general, SHP functions primarily as a transcriptional corepressor by interacting with various transcriptional factors, including nuclear receptors.(2) However, in this study, SHP was characterized as a positive regulator of bone differentiation, a finding that is inconsistent with its classic function. Among SHP target transcription factors, members of the PPAR family are known to be positive regulatory partners, and it has been reported that SHP is an endogenous enhancer of the transcriptional activity of PPARα(44) and that the activation of PPARγ is due in part to the inhibition of NCoR activity by SHP.(45) Previously, we have investigated the relation between SHP and PPARγ during adipogenic process in preadipocyte 3T3-L1. However, the expression of SHP was decreased during adipogenesis (data not shown). Thus this result suggests that SHP may not be involved in adipogenesis in connection with PPARγ. Because SHP does not possess a DNA-binding domain, it may exert its role in bone formation through interaction with a specific coregulator. As a target regulator of SHP activity, we identified HDAC4, which blocks DNA binding of Runx2 to its target gene promoter. Our finding suggests that SHP uses a competition mechanism to regulate bone metabolism, which is similar to the regulation mechanism of SHP on PPARγ transactivity. Recently, it was reported that SHP increases gene expression of cyclooxygenase-2 (COX-2) via the sequential transcriptional induction of a caudal-related homeobox gene (*CDX1*) in human gastric cancer cells.(46) With regard to its controversial regulatory propensity, these reports suggest that SHP may participate differently in various cellular functions depending on the cell type. Previous reports demonstrated that *HDAC4*^−/−^ mice exhibit a phenotype similar to that observed in mice with constitutive expression of Runx2 in chondrocytes, and HDAC4, which is expressed in prehypertrophic chondrocytes, regulates chondrocytes hypertrophy and endochondral bone formation by inhibiting the Runx2-binding activity.(40) In fact, endochonral bone formation begins with condensation of mesenchymal cells and their subsequent differentiation into chondrocytes, which organize into a template of the eventual bone comprised of a succession of proliferating, prehypertrophic, and hypertrophic regions.(47) As shown in Figure 5F, SHP^−/−^ mice showed considerably fewer chondrocytes than WT mice. Thus our results also support the hypothesis that the transactivity of Runx2 is regulated by HDAC4 and that inhibition of HDAC4 promotes osteoblast differentiation and bone formation.

This study presents new evidence that the orphan nuclear receptor SHP is a novel positive regulator of osteoblast differentiation and bone formation. These findings may open new areas for exploring the molecular mechanisms that underlie osteoblast differentiation by orphan nuclear receptors. Further, these findings also may contribute to the development of therapies based on the manipulation of SHP as an approach in the treatment of metabolic bone diseases such as osteoporosis. As of now, a natural ligand of SHP has not been identified, although one research group has proposed adamantyl-substituted retinoid-related molecules as potent regulators of SHP.(48) Thus it is important to pursue the identification of specific ligand(s) or agonist(s) of SHP, the identity of which will provide valuable tools for controlling bone metabolism.
